# Development of the full-length cDNA clones of two porcine epidemic diarrhea disease virus isolates with different virulence

**DOI:** 10.1371/journal.pone.0173998

**Published:** 2017-03-16

**Authors:** Jie Li, Zhonghui Jin, Yueyi Gao, Lei Zhou, Xinna Ge, Xin Guo, Jun Han, Hanchun Yang

**Affiliations:** Key Laboratory of Animal Epidemiology and Zoonosis of Ministry of Agriculture, College of Veterinary Medicine and State Key Laboratory of Agrobiotechnology, China Agricultural University, Beijing, People’s Republic of China; Sun Yat-Sen University, CHINA

## Abstract

The recently emerged highly virulent variants of porcine epidemic and diarrhea virus (PEDV) remain a huge threat to the worldwide swine industry. Here, we describe the development of a bacterial artificial chromosome (BAC) reverse genetics system for PEDV based on two recent Chinese field isolates, namely CHM2013 and BJ2011C. Phylogenetically, CHM2013 is closely related to the vaccine strain SM98 whereas the isolate BJ2011C belongs to the GIIb group, a cluster that contains many recent pandemic strains. The full-length cDNA clones of the two isolates were constructed into BAC under the control of CMV promoter. The rescued viruses rBJ2011C and rCHM2013 were found to replicate at the kinetics similar to their respective parental viruses in cell culture. When tested in the 2-day-old pig model, rBJ2011C caused severe diarrhea of piglets with extensive damages to the intestinal epithelium, leading to 100% fatality within 48 hours. In contrast, the rCHM2013-inoculated piglets all survived with only very minor tissue damage observed. Thus, we have successfully established a convenient platform for PEDV genome manipulation. This study also represents the first description of a DNA-launched reverse genetics system for the highly virulent PEDV.

## Introduction

Porcine epidemic diarrhea virus (PEDV) is an economically important pathogen of swine; it mainly causes porcine epidemic diarrhea (PED), a disease that is characterized by acute enteritis, diarrhea, vomiting and dehydration [1–5]. In the field, PEDV can infect pigs of all ages, but the highest mortality often occurs to the newborns of one week old [6–10]. As a positive-stranded RNA virus, PEDV belongs to the genus *Alpha-Coronavirus* within the family *Coronaviridae* in the order *Nidovirales* [8, 11, 12]; it has a genomic size of about 28 kb that contains at least 7 ORFs. Of them, ORF1a and ORF1b encode replicase proteins important for viral replication and anti-host immunity, whereas other five ORFs code for structural/accessory proteins, including spike protein (S), ORF3, envelope (E), matrix protein (M) and nucleocapsid protein (N) [8, 11, 12].

The outbreak of PED can be dated back to early 1970s when England reported the first case in nursing piglets showing symptoms different from that of conventional transmissible gastroenteritis (TGE) [13]. The etiological agent (CV777) however was not identified until 1978 by a group of scientists from Belgium with the full-length genome eventually determined in 2001 [2, 11]. Subsequently, PEDV spread across Europe and to countries in Asia including South Korea and China [14–17]. During the two decades from 1990 to 2009, the disease generally occurred in a sporadic, infrequent manner with a low positive rate due to the vaccination intervention (e.g., CV777, SM98, and DR-13, etc.) [14, 18, 19]. In China, PEDV was identified for the first time around 1984 [20, 21]. The emergence of the highly pathogenic PED appeared to be sudden; it began in late 2010 and hit hard on the Chinese swine farms in large scale [22–24]. Three years later, it stroke North America and killed at least 8 million pigs within a very short period of time, leading to colossal economic losses [25–29].

The novel variants of PEDV are the major cause of the PED global pandemic; the epidemic viruses are mainly characterized by deletions, insertions or amino acid substitutions in the S gene and other regions as compared to the classical strains such as CV777 [4, 22, 30]. During the last 5 years, the field has accumulated substantial knowledge about the epidemiology and genetic evolution of the PEDV variants [3, 4, 8, 22–25, 31, 32], but understanding of the pathogenic mechanisms has been hindered by the difficulties in isolation and propagation of the epidemic viruses in culture as well as in virus genome manipulation. Nevertheless, progresses have been made, in particular on the latter [23, 33–35]. In 2013, Li et al reported the first genetic manipulation of PEDV genome by using targeted genetic recombination in mammalian cells [36]. Following that, Jengarn et al engineered the infectious cDNA clone of PEDV into the bacterial artificial chromosome (BAC) but based on a cell adapted G1 strain AVCT12 [34]. Most recently, two groups of scientists constructed the cDNA clones for the highly pathogenic PEDV strains of GIIa cluster (e.g., PC22A and AH2012/12); However, the full-length cDNA genomes were assembled by *in vitro* ligation of a set of contiguous cDNAs fragments of PEDV coupled with *in vitro* transcription to generate infectious viral RNA [33, 35].

To date, there is no report of a convenient platform for the highly virulent PEDV. In this paper, we describe such a reverse genetics system in BAC for the highly pathogenic PEDV of the GIIb group. In addition, we constructed the full-length cDNA clone of a low virulent Chinese isolate of PEDV. In both cases, the virus genome is subject to the control of the CMV promoter. This DNA-launched system allowed the successful recovery of infectious virions upon transfection of the recombinant BAC plasmids into Vero cells. Moreover, the infectious clone-derived viruses behaved just like their respective parental virus both *in vitro* and *in vivo*.

## Materials and methods

### Ethics statement

The animal experiments were performed according to the Chinese Regulations of Laboratory Animals—The Guidelines for the Care of Laboratory Animals (Ministry of Science and Technology of People’s Republic of China) and Laboratory Animal-Requirements of Environment and Housing Facilities (GB 14925–2010, National Laboratory Animal Standardization Technical Committee). The license number associated with this research protocol was CAU20160520-1, which was approved by the Laboratory Animal Ethical Committee of China Agricultural University.

### Cells, viruses, and antibodies

Vero CCL-81 cells (ATCC) were maintained in Dulbecco’s Modified Eagle Medium (DMEM) (Gibco) supplemented with 10% fetal bovine serum (Gibco) and 10 μg/ml Cellmaxin (GenDEPOT) at 37°C with 5% CO_2_. The PEDV strains BJ2011C and CHM2013 used this study were isolated on Vero CCL-81 cells from small intestine samples of pigs with PED symptoms. In addition, propagation of PEDV BJ2011C requires supplementation of trypsin at 10 μg/ml for optimal virion production. The mouse monoclonal antibody 4E3 was made by our lab using the recombinant N protein from PEDV strain BJ2011C as the immunogen that was expressed and purified from *E*.*coli* BL21 cells.

### Genome sequencing and phylogenetic analysis

Virion RNAs were extracted from PEDV-infected cell supernatant by using the QIAamp viral RNA kit (Qiagen) before being subject to cDNA synthesis by using MLV reverse transcriptase (promega). 27 sets of primers were designed to cover the full-length genome of PEDV, and the PCR products were gel purified and cloned into pGEM-T vector (Promega) with 3–4 clones chosen for sequencing. The sequences of 5’ and 3’ ends of PEDV genome were determined by SMART^™^ RACE cDNA amplification Kit (Clontech, CA). The phylogenetic trees were constructed using the neighbor-joining method of MEGA7. The phylogeny was tested using bootstrap methods with 1000 replications and the evolutionary distances were computed using the p-distance method. All positions with less than 95% site coverage were eliminated. That is, fewer than 5% alignment gaps, missing data, and ambiguous bases were allowed at any position.

### Construction of PEDV full-length cDNA clone in BAC

The PEDV BJ2011C passage 8 (P8) and CHM2013 passage 4 (P4) were used for construction of infectious cDNA clone in a BAC system. To assemble the full-length cDNA of BJ2011C, the genome was divided into 6 fragment (A: nt 1–2806, B: nt 2806–6808, C: nt 6808–13013, D: nt 13013–21039, E: nt 21039–26894, and F: 26894–28038). Due to the large size of segments C and D, we further divided C and D fragments into two parts (C1: nt 6808–9450, C2: 9450–13013; D1: 13013–16840; D2: 16840–21039) to minimize PCR-introduced mutation rate. In addition, the CMV promoter was added to the 5’end of fragment A by overlapping PCR so that the PEDV genome transcription will be under control of this promoter. To enhance the proper processing of PEDV genome mRNA, hepatitis delta virus ribozyme core sequence and bovine growth hormone (BGH) polyadenylation signal were added to the 3’ end of fragment F [37]. Each of the above fragments was PCR amplified, gel purified, and then cloned to pEASY-Blunt fast ligation vector (TransGen Biotech), and the correct clones were subsequently verified by DNA sequencing. The corresponding fragments were then PCR amplified and cloned into the BAC plasmid pBAC-I, an intermediate that has been modified based on plasmid pBleoBACII (NEB) to contain a restriction enzyme linker (NARI-FSEI-MLUI-PACI-APAI-SACII-ASCI-SPHI) to facilitate the assembly of PEDV full-length cDNA clone. Specifically, the fragments were assembled into the BAC plasmid step by step in an order of A-F-C-D-E-B by homologous recombination with the ClonExpress II One Step Cloning Kit (Vazyme). Each intermediate assembly was verified by DNA sequencing before proceeding to the next step, and the final plasmid was named pBAC-BJ2011C. The similar strategy was used to generate the full-length cDNA clone of CHM2013, namely the plasmid pBAC-CHM2013. The primers used for construction of PEDV infectious clones were listed in S1 Table.

### *In vitro* transfection

The recombinant BAC plasmids were prepared by using the Omega Maxi plasmid prep kit (Omega). The DNA was evaluated for quality on 0.8% agarose gel and quantified by spectrophotometry at the optical density at 260 nm (OD_260_). For transfection, Vero CCL81 cells were seeded into six-well plates in 2 ml of the complete medium containing 10% fetal bovine serum and then incubated at 37°C in 5% CO_2_ for 20 to 24 h until approximately 80% confluency. 1.2 μg of the recombinant BAC plasmids were transfected into Vero cells with the Attractene transfection reagent (Qiagen) by following the manufacture’s protocol. At 24 h post transfection, the cells were washed once and then replenished with serum-free DMEM. For the cells transfected with pBAC-BJ2011C, trypsin was added at a final concentration of 10 μg/ml to allow the virus recovery.

### Indirect immunofluorescence assay

The Vero CCL81 cells seeded in 96-well plates (COSTAR) were infected with passage 1 viruses of rBJ2011C and rCHM2013. At 24 h post infection, the cells were fixed with -20°C cold ethanol at room temperature for 10 min, followed by three washes with PBS. After being blocked with PBS containing 2% BSA, the cells were incubated with mouse monoclonal antibody 4E3 to PEDV N protein at a dilution of 1: 2, 000 at 37°C for 1 h. Followed by three washes with PBS for 5 min each, the cells were reacted with FITC-conjugated goat anti-mouse IgG at 37°C for 1 h. Nuclear DNA was stained with DAPI [Molecular Probes] for 5 min. After three rinses, the samples were mounted in PBS and examined under a Nikon inverted fluorescence microscope.

### Multi-step growth curve

Vero cells in 12-well plates were infected with the wild type or recombinant virus of PEDV at an MOI of 0.01. At different time points, the cell supernatants and the infected cells were collected, and the amount of total virus was quantified by the end-point dilution assay (TCID_50_).

### Viral plaque assay

Vero cells in six-well plates were infected with wild-type PEDV or the infectious clone-derived viruses at different dilutions. After absorption for 1h at 37°C, the unbound viruses were removed, and the cells were washed three times with DMEM before being overlaid with 1XDMEM containing 0.5% methylcellulose. For BJ2011C, trypsin was also added at a final concentration of 10 μg/ml trypsin. At 36 h post infection, the medium was removed and the plaques were stained with 3.7% paraformaldehyde containing 0.1% crystal violet.

### Animal experiments

Twelve 2-day-old piglets were randomly divided into 3 groups with 4 pigs for each group. The piglets were fed with a mixture of bovine colostrum powder and fresh liquid milk every 4 hours. Before challenge, the piglets were confirmed negative for PEDV, TGEV, PDCOV, rotavirus, PRRSV and PRV by using PCR. For the challenge experiment, pigs were orally inoculated with 2 ml of rBJ2011C or rCHM2013 at a dose of 1X10^5^ TCID_50_/ml. The control group was mock-infected with 2 ml DMEM. After challenge, rectal swabs were collected daily for measuring virus shedding in feces by Taqman real time RT-PCR targeting the conserved N gene of PEDV. The sequences were as follows: upstream primer: 5’-AATCCAGGGCCACTTCGAA-3’; downstream primer: 5’-TTCGCCCTTGGGAATTCTC-3’; Taqman Probe: 5’-AACGTGACCTCAAAGACATCCCAGAGTGG-3’.

The animals were monitored every 4 h for clinical signs of disease and weight gain. For the mock and CHM2013 groups, the piglets had been healthy all the time, and we used euthanasia as humane endpoints at 7 days post infection (dpi) by injecting pentobarbital sodium at a dose of 150 mg/kg via ear intravenous route. To reduce the stress to other animals, the euthanasia was carried out in a soundproof room to avoid panic of the rest living pigs. During the whole process, we tried out best to provide comfort for the piglets. For the BJ2011C group, we monitored the piglets for the following signs: severe diarrhea for more than 12 h, rejection for food for more than 8 h, and inability to move when we they were touched. When these three conditions are matched, euthanasia will be carried out as described above. Unfortunately, 2 piglets in this group died without euthanasia. One showed diarrhea for 8 h, refused food for 4 h, and died suddenly; the other could eat all the time, but suddenly died at 40 h post inoculation. At necropsy, different sections of intestinal tissues were collected for histopathological examination and stained with hematoxylin and eosin (H&E), and for quantitation of virus abundance by Taqman Real-Time RT-PCR as described above.

### Statistical analysis

The statistical analyses were performed by using a one-way or two-way RM ANOVA test in the GraphPad Prism 5 (CA, USA). The significance values were represented as follows: *or ^#^: P<0.05 (statistically significant); ** or ^##^: P<0.01; *** or ^###^: P<0.001 (extremely significant).

## Results

### Isolation and phylogenetic analysis of PEDV isolates BJ2011C and CHM2013

The initial outbreak of highly pathogenic PED took place around 2010 in South China and then quickly spread to other parts of the country [22, 23]. In 2011, we isolated a PEDV strain named BJ2011C from a pig farm in Beijing undergoing an acute PED outbreak, and then another strain named CHM2013 in 2013 from a PEDV vaccinated swine farm. In cell culture, both viruses were able to induce cell-cell fusion of Vero cells; however, propagation of PEDV BJ2011C was strictly trypsin-dependent. In animal studies, CHM2013 did not cause apparent clinical symptoms of 2-day-old piglets when orally inoculated at a dose of 2X10^5^ TCID_50_. In contrast, BJ2011C infections at the same dose led to 100% death of piglets within 48–72 hours accompanying severe diarrhea (S1A, S1B and S1C Fig). Thus, BJ2011C is a highly pathogenic isolate while CHM2013 is a low virulent virus.

**Fig 1:**
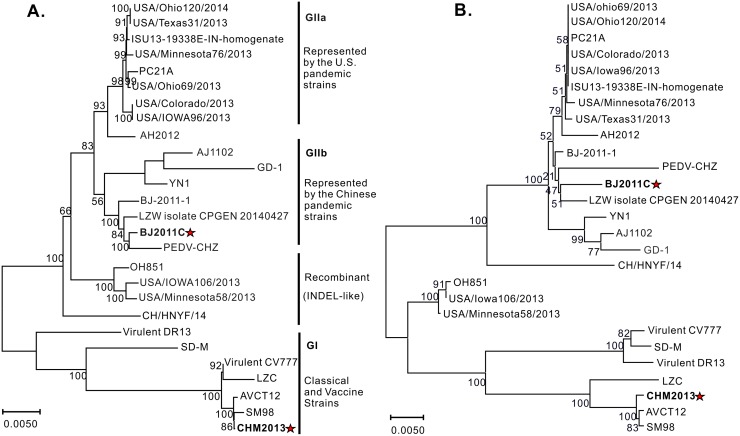
Genotyping of PEDV strains BJ2011C and CHM2013. (**A**) Genotyping of BJ2011C and CHM2013 based on the whole genome alignment; (**B**) Phylogenetic analysis based on S gene comparison. The tree was constructed with the neighbor-joining method (bootstrap n = 1000) based on the MEGA7 alignment of the respective nucleic acids or proteins. The tree was drawn to scale with branch lengths in the same units as those of the evolutionary distance used to infer the phylogenetic tree.

The full-length genomes of the two isolates were subsequently determined. BJ2011C was found to have a genomic size of 28,038 nt (GenBank accession no: KX066126) while CHM2013 was 27994 nt in length (GenBank accession no: KM887144.1), excluding the poly (A) tail. To understand the phylogeny, CHM2013 and BJ2011C were aligned against twenty-five other PEDV strains including the classical and recent variants, and the whole genome-based phylogenetic tree was drawn accordingly. As shown in Fig 1, the PEDV strains are mainly divided into two groups: G1 and G2 (Fig 1A). Specifically, CHM2013 belongs to the G1 group together with the classical PEDV strains CV777, AVCT12 and SM98 (Fig 1A), sharing identity ranging from 95.1% to 99.8 at nucleotide level. In contrast, BJ2011C belongs to the G2 group together with most of the current PEDV epidemic variants [1, 29, 38, 39]. Interestingly, BJ2011C is not grouped into the same clade as the U.S. strains or the Chinese strain AH2012, but instead exhibits a much closer genetic relationship with other Chinese strains such as BJ-2011-1, CHZ and YN1 (Fig 1A), indicating an apparently divergent evolution.

Compared to the classical strains of G1 group, the S protein of many pandemic viruses contain had a total of 5 amino acid deletion and 2 amino acid insertion. This feature was also observed in the S protein of PEDV BJ2011C strain. The S protein-based phylogenetic tree (Fig 1B) is slightly different from that based on whole genome alignment (Fig 1A). That is, BJ2011C was not grouped any more into the same clade as YN1, AJ1102 and GD-1; however, it consistently showed a close relationship with the strains BJ-2011-1, LZW and CHZ (Fig 1B). On the other hand, CHM2013 has a 22-nucleotide deletion at the end of S gene and a 30-nucleotide deletion at the start of ORF3, including the start codon ATG of ORF3. This deletion is not seen in strains such as CV777, but is consistent with that of SM98, AVCT12 and EASI strains. Of note, SM98 was a Korean strain while the viruses AVCT12 and EASI were of Thailand origin [34, 40, 41].

### Construction of the infectious cDNA clones of BJ2011C and CHM2013

**Fig 2:**
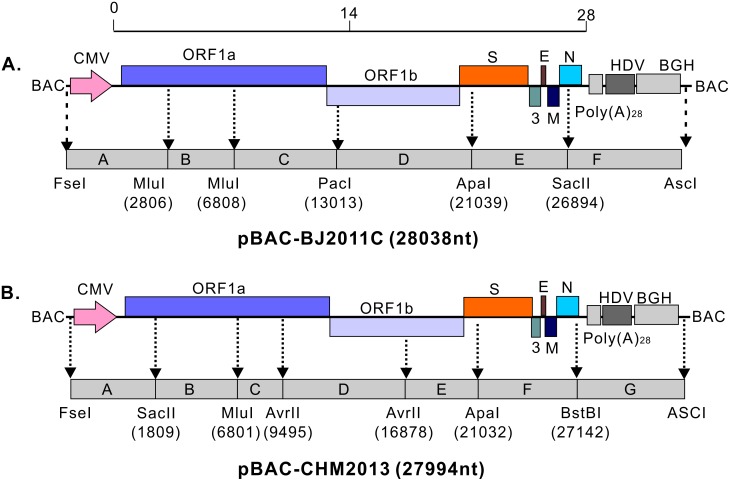
Construction of the full-length clones of PEDV strains BJ2011C and CHM2013 in the BAC system. The cDNA segments of PEDV BJ2011C (**A**) and CHM2013 (**B**) were cloned into the BAC plasmid to assemble the full-length cDNA clones. The PEDV cDNA genome was set under the control of the CMV promoter and followed by a HDV ribozyme sequence and a BGH polyadenylation signal. The restriction enzyme sites and the divided gene segments were also indicated that allowed the sequential assembly of a full-length cDNA clone in the BAC plasmid.

We used the BAC plasmid (pBeloBAC11) for assembly of the full-length cDNA clones of BJ2011C and CHM2013. In this system, the PEDV genome is subject to the control of the CMV promoter so that the viral mRNA can be synthesized by the host RNA polymerase II upon transfection of BAC plasmids into Vero cells. In addition, HDVRz and BGH sequences were added to the 3’ end of PEDV genome to aid the proper processing of a poly(A) tail [37, 42]. To facilitate amplification, the BJ2011C genome was divided into 6 segments (A, B, C, D, E and F) with the relative positions and the corresponding enzymes shown in Fig 2A. Of note, the 5’ end sequence in fragment A was fused to the CMV promoter while the viral 3’ end in fragment F was fused to the sequence containing PolyA (28 A), HdvRz and BGH polyadenylation signals. All DNA pieces were PCR amplified, ligated into pEasy-blunt vector, and then verified by DNA sequencing. A synonymous mutation (G15932A) was found in fragment D and retained as a genetic marker to distinguish from the parental virus. Meanwhile, to facilitate the construction, the BAC plasmid pBeloBAC11 was modified to create a shuttle plasmid pBAC-I that contains a set of multiple enzyme restriction sites as shown in Fig 2. Following that, each fragment was assembled into pBAC-I in an order of A-F-C-D-E-B by homologous recombination to generate the final plasmid pBAC-BJ2011C. Using the similar strategy, we engineered the BAC system for CHM2013 (Fig 2B) in which a synonymous mutation (T1809C) was introduced in segment A to serve as the genetic marker.

### Recovery and identification of recombinant viruses

**Fig 3:**
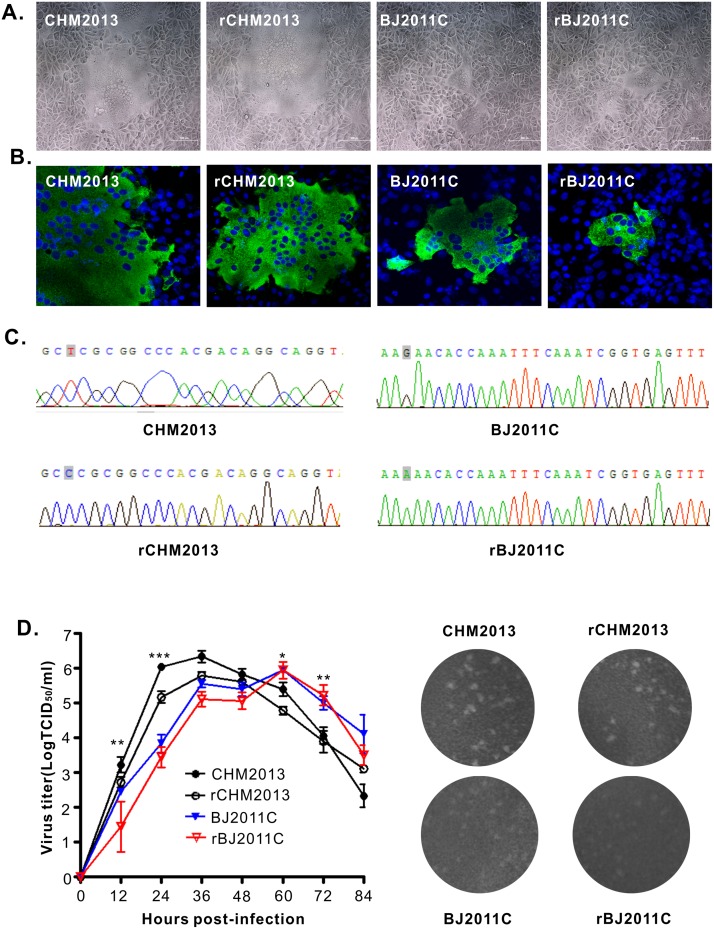
*In vitro* characterization of the infectious clone-derived viruses rBJ2011C and rCHM2013. (**A**) The virus-induced CPE at 24 h post infection (MOI = 0.01). (**B**) Indirect immunofluorescence staining of each virus with mouse monoclonal antibody 4E3 to PEDV N protein. (**C**) Detection of the genetic marker by sequencing of the recombinant viruses. (**D**) Multi-step growth curve of WT and recombinant viruses on Vero cells at an MOI of 0.01. (**E**) Comparison of plaque sizes and morphology of WT PEDV and the infectious clone-derived viruses on Vero cells at 4 days post infection. Asterisk (*) indicates a significant difference between rBJ2011C and rCHM2013 (*P<0.05; **P<0.01; ***P<0.001).

We transfected the plasmids pBAC-BJ2011C and pBAC-CHM2013 into Vero CCL81 cells at a confluency of 70–80% in six-well plates. For BJ2011C, the medium was changed to supplement trypsin at 24 h post transfection. The virus-induced CPE was monitored daily. After 48–96 h, the CPE appeared in the well transfected with pBAC-CHM2013 that was characterized by syncytium formation, which was similar to that observed for its parental virus CHM2013 (Fig 3A). For pBAC-BJ2011C, the CPE did not show up initially after transfection perhaps to low yield; however, it appeared upon passaged once on the new monolayers. The corresponding viruses were subsequently named rCHM2013 and rBJ2011C. The identity of the viruses was confirmed by the immunofluorescence assay using mouse monoclonal antibodies to PEDV N protein (Fig 3B) and by sequencing of the fragments carrying the genetic markers (Fig 3C). Thus, we have successfully rescued the recombinant PEDV viruses by using the DNA-launched system.

### *In vitro* growth properties of recombinant viruses

We next investigated the growth properties of the rescued viruses by the multi-step growth assay with an MOI of 0.01. As shown in Fig 3D, rCHM2013 and rBJ2011C exhibited growth kinetics similar to their respective parental viruses. However, at each time point, the titer of the recombinant virus was slightly lower than that of the parental virus. This is likely due to the fact that the parental viruses exist as quasi-species while the infectious clone-derived viruses represent relatively a homogeneous population. In addition, the results from the plaque assay showed that the rescued viruses had the similar size and plaque morphology to that of their respective parental virus (Fig 3E). On the other side, rBJ2011C generally made smaller plaques than that of rCHM2013, and this result is consistent with the observation that rBJ2011C had a slower growth kinetics as compared to rCHM2013. Nevertheless, both viruses could reach peak titers over 10^5^ TCID_50_/ml in cell culture.

### Pathogenicity analysis

**Fig 4:**
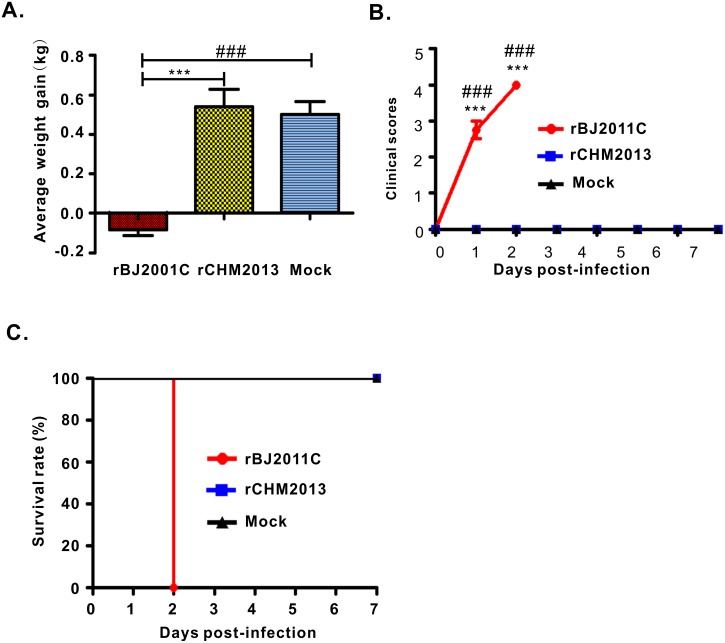
Pathogenicity analyses of PEDV rBJ201C and rCHM2013. (**A**) The average weight gain of piglets at the day of death or euthanasia at the final time points. (**B**) The clinical mental state scores of piglets of different groups. Criteria for evaluation: 0 = normal; 1 = mild lethargy (slow to move, head down); 2 = moderate lethargy (stands but tends to lie down); 3 = heavier lethargy (lie down, occasionally stand); 4 = severe lethargy (recumbent, moribund). (**C**) The survival rate of piglets of each group. Asterisk (*) indicates a significant difference between rBJ2011C and rCHM2013 (***P<0.001). Pound (#) indicates a significant difference between rBJ2011C and Mock (^###^P<0.001).

We next tested the pathogenicity of the rescued viruses rBJ2011C and rCHM2013. To do that, twelve 2-day-old piglets were randomly divided into three groups and orally inoculated with rCHM2013 or rBJ2011C at a dose of 2×10^5^ TCID_50_ or mock-infected with DMEM. Overall, the rescued viruses (rBJ2011c and rCHM2013) recapitulated the features in aspects of virulence and clinical symptoms of their respective parental viruses aforementioned (Fig 4 and S1 Fig). Specifically, for the rBJ2011C group, one piglet started severe diarrhea at 24 h post challenge, and the other three developed severe diarrhea around 30 h after challenge. Overall, all four piglets exhibited substantial weight loss (Fig 4A), had the highest clinical score (Fig 4B), and died within 48 hours (Fig 4C), indicating the highly virulent nature of rBJ2011C. In contrast, there was only very mild diarrhea observed for 2 piglets from the mock group and 1 from rCHM2013 group on day 1 post challenge (Fig 4B). In addition, this symptom last for only 24 h. Other than that, these two groups of piglets stayed in good appetite and showed stable weight gain (Fig 4A).

**Fig 5:**
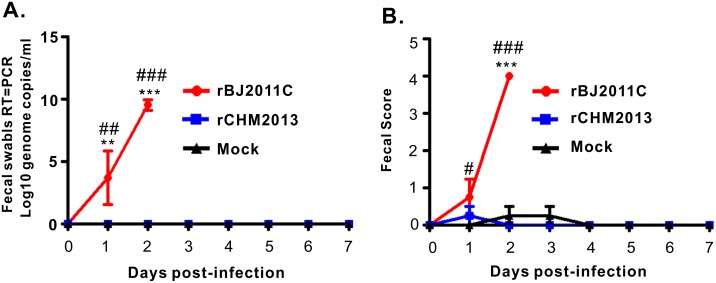
Virus shedding in feces and fecal scores. (**A**) Virus load in feces as measured by Real-Time RT-PCR detection of virus genome in fecal swabs. (**B**) Fecal scores. Evaluation standard: 0 = normal; 1 = soft (cowpie); 2 = very soft and tend to be liquid 3 = liquid with some solid content; 4 = watery diarrhea with no solid content. Asterisk (*) indicates a significant difference between rBJ2011C and rCHM2013 (**P<0.01; ***P<0.001). Pound (#) indicates a significant difference between rBJ2011C and Mock (^#^P<0.05; ^###^P<0.001).

We also investigated the virus shedding in the feces by real-time PCR. It was found that two piglets from the rBJ2011C group started the virus shedding at a high level within 24 h post challenge (Fig 5A), and all four shed the virus within 36–48 h post challenge (Fig 5A). In contrast, no detectable virus shedding could be found for the mock or rCHM2013 group (Fig 5A). Consistently, the fecal score for rBJ2011C group is much higher than that for the rCHM2013 group (Fig 5B).

**Fig 6:**
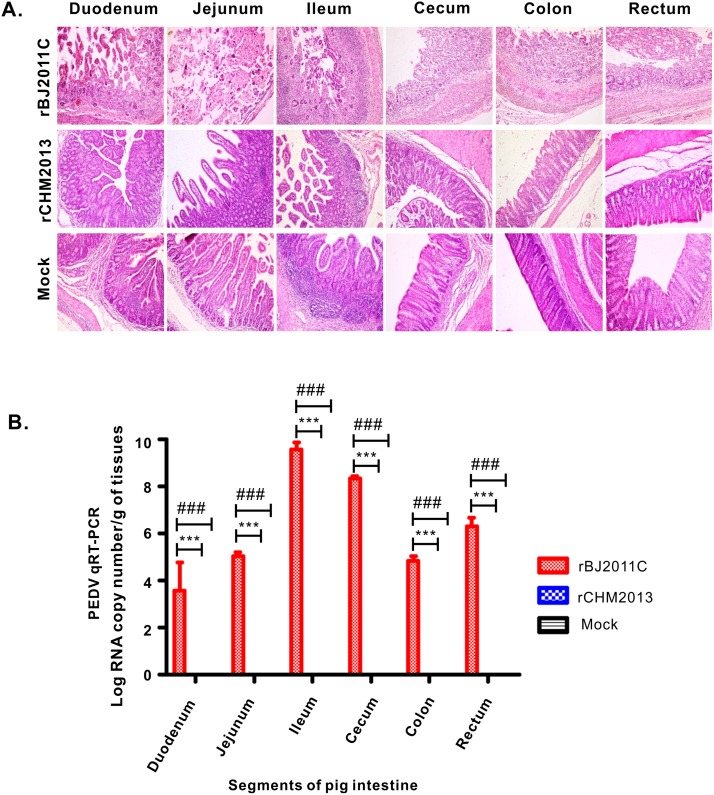
Histopathological examination of intestines of recombinant PEDV-infected piglets and quantification of viral load in different intestine segments. (**A**) Different segments, including duodenum, jejunum, cecum, colon and rectum, of intestines from each group were taken and then processed for HE staining, and the representative pictures were shown here. (B) Quantitation of viral load in different parts of intestine by TaqMan Real-Time RT-PCR targeting PEDV N gene. Asterisk (*) indicates a significant difference between rBJ2011C and rCHM2013 (***P<0.001). Pound (#) indicates a significant difference between rBJ2011C and mock (^###^P<0.001).

Finally, we examined the histopathology and viral load in the intestinal tract. Basically, different segments of intestine, including duodenum, ileum, jejunum, cecum, colon and rectum, were taken and individually processed for HE staining and for quantification of virus abundance. As shown in Fig 6A, infection by rBJ2011C caused extensive damages to all intestinal segments characterized by large amounts of fragmentation and shedding of intestinal villi. The epithelial layer of intestines was destroyed and lost the integrity; the most severe damages occurred in Jejunum. In contrast, the damages caused by rCHM2013 were mainly restricted to Jejunum and Ileum, which were quite minor as compared to the rBJ2011C group. Consistent with the histopathological analyses, the quantitative RT-PCR results showed that rBJ2011c infected all segments of intestine. The ileum apparently had the highest viral load; however, the virus abundance in the Jejunum was several logs lower. This was likely due to the severe damage of Jejunum epithelial layer as a result of virus infection. On the other hand, no viral RNA could be detected in the CHM2013 group.

## Discussion

In this paper, we reported the isolation and characterization of two Chinese PEDV field isolates. In particular, CHM2013 is a low virulent virus that is closely related to the Korean vaccine SM98. In contrast, the strain BJ2011C is highly pathogenic and belongs to the GIIb group, a cluster that is associated with PED pandemic. Most importantly, we successfully established a DNA-launched BAC system for both viruses.

Coronaviruses are enveloped, positive-stranded RNA viruses [12]. Selective pressures from vaccination and environmental changes have led to the emergence of novel virulent variants of this class of viruses, such as SARS-CoV and the highly pathogenic PEDV (HP-PEDV), which pose great threat to the health of both humans and animals [22, 29, 43]. The emergence of HP-PEDV was sudden and occurred around 2010 in China [22, 23], followed by the devastating PED outbreak on the North American continent three years later [26, 29]. Phylogenetic analyses revealed that the earlier Asian isolates such as the Chinese AH2012 and Korean KNU-1305 share high nucleotide identity with the North American prototype strains and therefore are clustered within the GIIa group, suggesting a common evolutionary origin and a possible transmission route from Asia to North America [25, 26, 29, 44]. Interestingly, the PEDV strain BJ2011C in this study is not clustered in the same branch as the AH2012 but rather grouped to GIIb cluster (Fig 1). The S genes from both GIIa and GIIb groups contain the similar gene deletion and insertion pattern as reported before, suggesting that they are from a common ancestor. However, the differences between the two groups suggest that they separated at some time point to undergo independent evolution that led to accumulation of additional mutations. Although deletion and insertions are the characteristic features for GIIa and GIIb strains, whether they contribute to PEDV pathogenesis have remained unclear. In addition, some laboratory and field strains contain large deletions in S gene [32, 45, 46]. However, some study showed that the strain that contains the large deletion in S gene was mild to piglets, suggesting that the changes of S gene of PEDV might be closely tied to the variations of pathogenicity [32, 45, 46]. The exact causative relationship between S size variation and the pathogenicity is still waiting to be determined.

Reverse genetics provides a very powerful tool for studying viral pathogenesis and for developing new generation of vaccines for disease control. Due to the large size of coronaviruses and other hurdles, it has been challenging to establish such a system. For PEDV, several approaches have been tried with different degrees of success [33–36]. Li et al reported successful recovery of infectious viruses by targeted RNA recombination strategy [36]. In this approach, the purified viral RNA is cotransfected into selective mammalian cells with a RNA fragment generated by in vitro transcription carrying two arms homologous to the virus. The host recombination machinery then mediates the homology recombination between the viral genomic RNA and the RNA fragment to generate a chimeric virus [36]. In general, the recombination efficiency is very low, and this approach can also select for viral mutants. Another approach is the assembly of the full-length PEDV genome by *in vitro* ligation [33, 35]. Basically, the PEDV genome is divided into several fragments. These cDNA fragments are PCR amplified, purified and then ligated *in vitro* to assemble the full-length cDNA clone. In this system, a T7 promoter is usually placed ahead of the 5’end of PEDV genome or a SP6 promoter is set at the 3’end of the virus genome; the *in vitro* transcription with the corresponding commercial kits will lead to transcription of the full-length viral mRNA, which is then introduced into mammalian cells by transfection [47, 48]. Two groups of scientists have recently used this method to successfully produce infectious cDNA clones for the highly pathogenic PEDV strains (e.g., PC22A and AH2012/12) [33, 35]. It should be noted that, however, this method is quite labor extensive, time-consuming and relatively inefficient, although representing a step forward compared to targeted genetic recombination.

The third approach is the BAC-based DNA-launched system. This is the most commonly used method for viruses with large genomes such as herpesviruses [49]. That is, the virus genome is cloned into BAC and set under control of the CMV promoter. Upon transfection of the PEDV-containing BAC plasmid into eukaryotic cells, the viral mRNA will be synthesized by host RNA polymerase II. Certainly, the most challenging problem is the stability issue in *E*.*coli* of the BAC plasmid perhaps arising from the toxicity of the PEDV gene or gene products due to basal level of leaky expression [37, 42]. Nevertheless, Jengarn et al have recently managed to establish such a system for a cell adapted strain AVCT12 [34]. AVCT12 is a Thailand PEDV strain sharing high homology with CHM2013; they all have gene deletion at the end of S gene and the start ORF3 and this deletion leads to the inhibition of ORF3 expression. However, AVCT12 requires expression of APN in Vero E6 cells to make high yield [34]. In this study, in addition to a low pathogenic strain, we for the first time constructed the DNA-launched BAC system for the highly pathogenic PEDV strain. We used *E*.*coli* trans10 cells to improve the stability of the PEDV-containing BAC plasmids during propagation, and found that the BAC plasmids are very stable in these cells. In addition, VERO CCL81 cells were used for transfection and virus propagation. Different from AVCT12, CHM2013 was found to replicate well in both VERO-CCL81 and VERO-E6 cells without the need of supplementation of APN. BJ2011C is a high pathogenic strain and most closely related to the Chinese pandemic strain PEDV-CHZ. Through animal experiments, we confirmed the pathogenic nature of rBJ2011C. Thus, our studies not only fulfilled Koch’s postulations, but also provide BJ2011C as a good model organism to study the pathogenic mechanisms of highly virulent PEDV. With the reverse genetics system at hand for both high and low virulent strains, this should aid the construction of chimeric viruses for dissecting viral virulence determinants and facilitate genetic engineering of better vaccines.

## Supporting information

S1 Fig Pathogenicity analyses of PEDV BJ2011C and CHM2013. (**A**) The clinical mental state score of piglets. Criteria for evaluation: 0 = normal; 1 = mild lethargy (slow to move, head down); 2 = moderate lethargy (stands but tends to lie down); 3 = heavier lethargy (lie down, occasionally stand); 4 = severe lethargy (recumbent, moribund). (**B**) Fecal scores. Evaluation standard: 0 = normal; 1 = soft (cowpie); 2 = very soft and tend to be liquid; 3 = liquid with some solid content; 4 = watery diarrhea with no solid content. (**C**) The piglet survival rate. Asterisk (*) indicates a significant difference between BJ2011C and CHM2013 (***P<0.001). Pound (#) indicates a significant difference between rBJ2011C and Mock (###P<0.001).
(TIF)

S1 Table Primers used for construction of infectious cDNA clone of PEDV. (XLSX)
